# Supplementary material for: Genome-Wide Association Study Identifies Candidate Genes Related to the Linoleic Acid Content in Soybean Seeds
Source: Int J Mol Sci. 2021 Dec 31;23(1):454. doi: 10.3390/ijms23010454 (PMC8745128; doi:10.3390/ijms23010454)
Supplement: Supplementary file 1 [file ijms-23-00454-s001.zip › Figure S2.pdf]

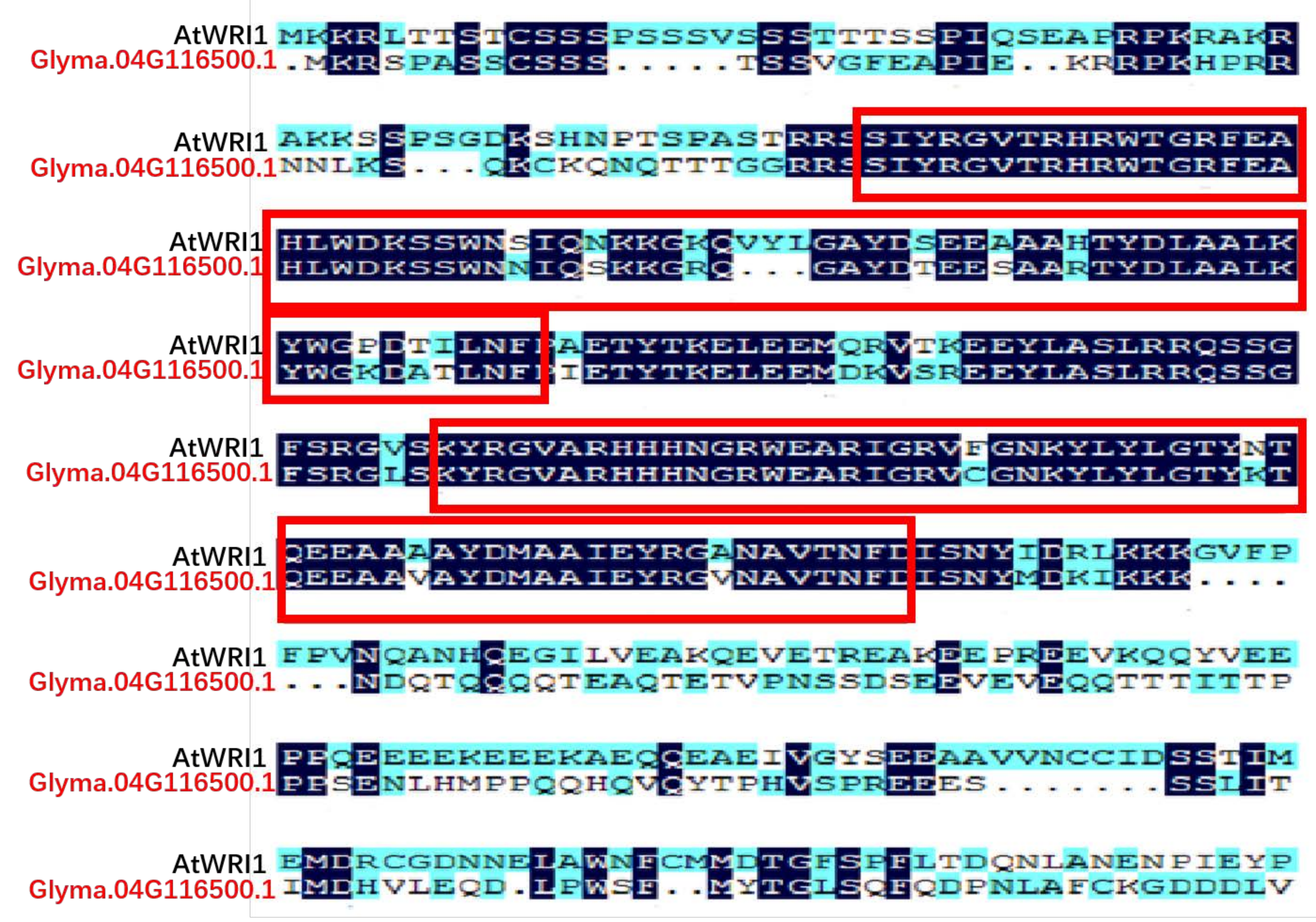
AtWRI1 MKKRLTTSTCSSSPSSSVSSSTTTSSPIQSEAPRPKRAKR
Glyma.04G116500.1 .MKRSPASSCSSS.....TSSVGFEAPIE..KRRPKHPRR
AtWRI1 AKKSSPSGDKSHNPTSPASTRRSSIYRGVTRHRWTGRFEA
Glyma.04G116500.1 NNLKS...QKCKQNQTTTGGRRSSIYRGVTRHRWTGRFEA
AtWRI1 HLWDKSSWNSIQNKKGKQVYLGAYDSEEAAAHTYDLAALK
Glyma.04G116500.1 HLWDKSSWNNIQSKKGRQ...GAYDTEESAARTYDLAALK
AtWRI1 YWGPDTILNFPAETYTKELEEMQRVTKEEYLASLRRQSSG
Glyma.04G116500.1 YWGKDATLNFPIETYTKELEEMDKVSREEYLASLRRQSSG
AtWRI1 FSRGVSKYRGVARHHHNGRWEARIGRVFGNKYLYLGTYNT
Glyma.04G116500.1 FSRGLSKYRGVARHHHNGRWEARIGRVCGNKYLYLGTYKT
AtWRI1 QEEAAAAYDMAAIEYRGANAVTNFDISNYIDRLKKKGVFP
Glyma.04G116500.1 QEEAAVAYDMAAIEYRGVNAVTNFDISNYMDKIKKK....
AtWRI1 FPVNQANHQEGILVEAKQEVETREAKEEPREEVKQQYVEE
Glyma.04G116500.1 ...NDQTQQQQTEAQTETVPNSSDSEEVEVEQQTTTITTP
AtWRI1 PEQEEEEKEEEKAEQCEAEIVGYSEEAAVVNCCIDSSTIM
Glyma.04G116500.1 PESENLHMPPQQHQVQYTPHVSPREEES........SSLIT
AtWRI1 EMDRCGDNNELAWNFCMMDTGFSPFLTDQNLANENPIEYP
Glyma.04G116500.1 IMDHVLEQD.LPWSE..MYTGLSQFQDPNLAFCKGDDDLV
