# Supplementary material for: Genome-Wide Association Study Identifies Candidate Genes Related to the Linoleic Acid Content in Soybean Seeds
Source: Int J Mol Sci. 2021 Dec 31;23(1):454. doi: 10.3390/ijms23010454 (PMC8745128; doi:10.3390/ijms23010454)
Supplement: Supplementary file 1 [file ijms-23-00454-s001.zip › Figure S4.pdf]

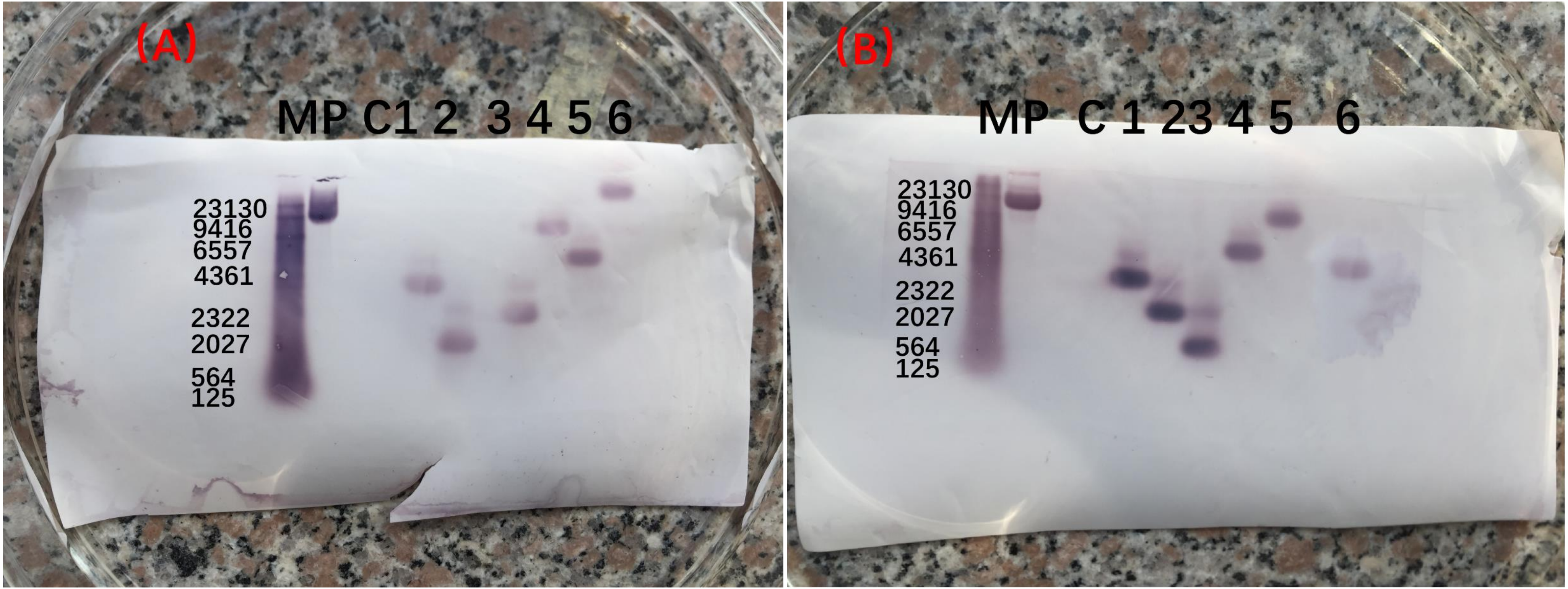

Figure S2. Verification of GmWRI14 expression in T0/T1 transgenic soybeans using Southern blot analysis. (A) Southern blot analysis of the copy number of the GmWRI14 expression cassette in T0 plants. (B) Copy number of the GmWRI14 expression cassette in T1 plants. C: control JN38. M: marker.
