# Supplementary material for: Genome-Wide Association Study Identifies Candidate Genes Related to the Linoleic Acid Content in Soybean Seeds
Source: Int J Mol Sci. 2021 Dec 31;23(1):454. doi: 10.3390/ijms23010454 (PMC8745128; doi:10.3390/ijms23010454)
Supplement: Supplementary file 1 [file ijms-23-00454-s001.zip › Table S1.pdf]

Table S1. Candidate genes related to linoleic acid in 2018-2020 years

Year	chromosome	Gene	Predicted Function	Length	Contribution rate
2018	Chr03	*Glyma.03G144500.1*	3PREDICTED: fatty acid desaturase-2, FAD2	1217	0.10
	Chr04	*Glyma.04G191100.1*	3PREDICTED: Glycine max probable pectate lyase 18-like (LOC100814679), mRNA	1657	0.32
2018 2019 2020		*Glyma.04G116500.1 (GmWRI14)*	3PREDICTED: plant WRI1 protein family	1820	0.63
		Glyma.04G203200.1	3PREDICTED: Glycine max respiratory burst oxidase homolog protein C-like (LOC100800248), mRNA	2440	0.08
		Glyma.04G084200.1	5PREDICTED: Glycine max probable transcriptional regulatory protein At2g25830-like (LOC100818936), transcript variant X2, mRNA	1213	0.11
	Chr07	*Glyma.07G033100.1*	3PREDICTED: Glycine max ADP,ATP carrier protein 1, chloroplastic-like (LOC100793284), mRNA	2317	0.12

	Glyma.07G089000.1	3PREDICTED: Glycine max VIN3-like protein 1-like (LOC100780157), transcript variant X2, mRNA	2756	0.10
Chr08	*Glyma.08G019700.1*	3PREDICTED: Glycine max calcium-dependent protein kinase 3-like (LOC100777096), transcript variant 1, mRNA	1877	0.16
	Glyma.08G185000.2	3PREDICTED: Glycine max probable plastid-lipid-associated protein 4, chloroplastic-like (LOC100803367), transcript variant 1, mRNA	979	0.15
Chr11	*Glyma.11G229600.1*	3PREDICTED: Glycine max DNA replication complex BAG protein，transcript variant 2, mRNA，	1257	0.47
Chr13	*Glyma.13G163400.1*	3PREDICTED: Glycine max protein S-acyltransferase 24-like (LOC100777470), misc_RNA	2490	0.32
Chr15	*Glyma.15G117700.1*	3PREDICTED: Glycine max uncharacterized LOC102666654 (LOC102666654), mRNA	693	0.17
	Glyma.15G120100.1	3PREDICTED: Glycine max tRNA methyltransferase 10 homolog A-like (LOC100779099), mRNA	1337	0.10
	Glyma.15G120200.2	3PREDICTED: Glycine max uncharacterized LOC102665381 (LOC102665381), mRNA	1227	0.08
	Glyma.15G127500.1	3PREDICTED: Glycine max polygalacturonase-like (LOC100785701), mRNA	1551	0.10

		Glyma.15G201700.1	3PREDICTED: Glycine max uncharacterized LOC100814752 (LOC100814752), mRNA	1945	0.11
		Glyma.15G244000.1	3PREDICTED: Glycine max uncharacterized LOC100814749 (LOC100814749), mRNA	1213	0.10
2019	Chr19	*Glyma.19G110600.1*	3PREDICTED: Glycine max uncharacterized LOC102659858 (LOC102659858), mRNA	1709	0.10
	Chr02	*Glyma.02G220300.1*	2PREDICTED: Glycine max ataxin-2-like (LOC100788042), mRNA	1135	0.18
	Chr03	*Glyma.03G144500.1*	3PREDICTED: fatty acid desaturase-2, FAD2	1217	0.30
2019	Chr04	*Glyma.04G116500.1 (GmWRI14)*	2PREDICTED: Glycine max uncharacterized LOC102660202 (LOC102660202), plant WRI1 protein family	1820	0.40
		Glyma.04G110500.1	5PREDICTED: Glycine max uncharacterized LOC100500088 (LOC100500088), transcript variant X1, mRNA	979	0.33
	Chr08	*Glyma.08G071600.1*	2PREDICTED: Glycine max metacaspase-3-like (LOC100796113), transcript variant X2, mRNA	1839	0.12
	Chr11	*Glyma.11G229600.1*	3PREDICTED: Glycine max DNA replication complex BAG protein, transcript variant 2, mRNA,	1257	0.47
	Chr12	*Glyma.12G224000.1*	2PREDICTED: Glycine max uncharacterized LOC102660202 (LOC102660202), mRNA	2799	0.17
		Glyma.12G227300.1	2PREDICTED: Glycine max DNA ligase 1-like (LOC100818049), mRNA	2728	0.17

	Chr20	*Glyma.20G111000.7*	2PREDICTED: Fatty acid desaturase-2, FAD2	896	0.08
2020	Chr03	*Glyma.03G054100.1*	3PREDICTED: Glycine max pleiotropic drug resistance protein 1-like (LOC100791601), mRNA	4662	0.41
		Glyma.03G163500.1	3PREDICTED: Glycine max probable pectate lyase 18-like (LOC100814679), mRNA	1657	0.23
		Glyma.03G168200.3	3PREDICTED: Glycine max respiratory burst oxidase homolog protein C-like (LOC100800248), mRNA	2440	0.38
	Chr04	*Glyma.04G191100.1*	3PREDICTED: Glycine max serine/threonine-protein phosphatase PP2A catalytic subunit-like (LOC100782279), mRNA	1655	0.33
		Glyma.04G203200.1	3PREDICTED: Glycine max ADP,ATP carrier protein 1, chloroplastic-like (LOC100793284), mRNA	2317	0.21
2020	Chr04	*Glyma.04G116500.1 (GmWRI14)*	2PREDICTED: Glycine max uncharacterized (LOC102660202) (LOC102660202), mRNA	1820	0.50
	Chr05	*Glyma.05G155300.1*	3PREDICTED: Glycine max DNA replication complex GINS protein PSF3-like (LOC100797609), transcript variant 2, mRNA	979	0.42
	Chr07	*Glyma.07G033100.1*	3PREDICTED: Glycine max protein S-acyltransferase 24-like (LOC100777470), misc RNA	1257	0.41
		Glyma.07G089000.1	3PREDICTED: Glycine max abscisic-aldehyde oxidase-like (LOC100812604), mRNA	2490	0.45

Cho19	*Glyma.19G110600.1*	3PREDICTED: Glycine max tRNA methyltransferase 10 homolog A-like (LOC100779099), mRNA	1337	0.29
