# Supplementary material for: Genome-Wide Association Study Identifies Candidate Genes Related to the Linoleic Acid Content in Soybean Seeds
Source: Int J Mol Sci. 2021 Dec 31;23(1):454. doi: 10.3390/ijms23010454 (PMC8745128; doi:10.3390/ijms23010454)
Supplement: Supplementary file 1 [file ijms-23-00454-s001.zip › Table S2.pdf]

Table S2. A Variance analysis of *GmWRI14* expression in different tissues of soybean

| Group | Linoleic acid content | | Name | relative expression in leaves | | relative expression in stem | | relative expression in roots | | relative expression in seed | |
|---|---|---|---|---|---|---|---|---|---|---|---|
| | mean | Sig. | | Mean | Sig. | mean | Sig | mean | Sig. | mean | Sig. |
| **(A)**Soybean lines with low linoleic acid | 34.23 | B | YR063 | 4.12 | d | 25.12 | b | 22.24 | b | 46.23 | ab |
| | | | YR004 | 4.23 | d | 24.23 | b | 23.79 | b | 45.80 | ab |
| | | | YR007 | 4.62 | d | 23.22 | b | 21.21 | b | 45.23 | ab |
| | | | YR016 | 4.12 | d | 26.21 | a | 19.21 | c | 52.25 | a |
| | | | YR032 | 4.25 | d | 25.22 | b | 26.42 | b | 52.32 | a |
| | | | YR030 | 3.22 | d | 28.25 | a | 30.13 | b | 45.42 | ab |
| | | | YR024 | 3.62 | d | 28.22 | a | 31.12 | b | 46.23 | ab |
| **(B)**Soybean lines with high | 66.23 | A | t6316 | 0.87 | e | 12.12 | d | 16.60 | c | 30.26 | b |
| | | | T6030 | 1.23 | e | 13.12 | d | 17.45 | c | 29.75 | b |
| | | | T6049 | 1.22 | e | 12.22 | d | 18.32 | c | 26.12 | b |

| | | | | | | | | | |
|---|---|---|---|---|---|---|---|---|---|
| linoleic acid | T6098 | 1.45 | e | 11.12 | d | 18.72 | c | 28.12 | b |
| | t6156 | 1.43 | e | 12.21 | d | 17.99 | c | 28.22 | b |
| | T6033 | 1.41 | e | 14.43 | d | 18.11 | c | 29.12 | b |
| | T6142 | 1.42 | e | 12.52 | d | 18.23 | c | 28.81 | b |

Note: The different uppercase letters indicate significant differences at *P < 0.01*, the different lower letters indicate significant differences at *P < 0.05*, as determined by Duncan's multiple-range test

Table S2. B The Correlation between the *GmWRI14* expression and linoleic acid content

| | | linoleic acid content | Expression in leaves | Expression in stems | Expression in roots | Expression in seed |
|---|---|---|---|---|---|---|
| linoleic acid content | Pearson relative | 1 | -0.901** | -0.910** | -0.906** | -0.912** |
| Expression in leaves | Pearson relative | -0.901** | 1 | 0.911** | 0.897** | 0.811** |
| Expression in stems | Pearson relative | -0.910** | 0.911** | 1 | 0.827** | 0.818** |

| | | | | | | |
|---|---|---|---|---|---|---|
| Expression in roots | Pearson relative | -0.906** | 0.897** | 0.827** | 1 | 0.854** |
| Expression in seed | Pearson relative | -0.912** | 0.811** | 0.818** | 0.854** | 1 |
