# Supplementary material for: Genome-Wide Association Study Identifies Candidate Genes Related to the Linoleic Acid Content in Soybean Seeds
Source: Int J Mol Sci. 2021 Dec 31;23(1):454. doi: 10.3390/ijms23010454 (PMC8745128; doi:10.3390/ijms23010454)
Supplement: Supplementary file 1 [file ijms-23-00454-s001.zip › Table S3.pdf]

Table S3. Fatty acid content and agronomic characters of *GmWRI14* transgenic soybean

Name	Oil content	Stearic acid	Palmitic acid	Oleic acid	Linoleic acid	Linolenic acid	Pods per plant	Seed number per plant	hundred-seed weight (g)	seed weight per plant (g)	Average plant height (cm)	Plot yield (g)
Control JN 38	24.23±1.1	8.94±2.1	2.65±0.1	24.37±2.5	68.18±2.2	3.68±0.2	32A	110A	10.91A	16.4a	106.292A	424.84A
JN38-GmWRI14-1	44.33±1.2	12.58±1.5	11.70±0.2	39.23±1.1	34.22±1.6	12.39±0.2	38A	122A	24.71B	15.4a	106.221A	1319.80B
JN38-GmWRI14-2	43.47±2.2	14.20±3.1	11.38±2.3	41.48±3.2	34.06±2.2	13.46±2.5	30A	104A	19.43C	14.9a	106.123A	1207.28C
JN38-GmWRI14-3	34.46±3.1	12.30±2.1	11.95±2.6	39.54±2.1	33.43±2.4	11.10±1.3	32A	108A	19.82C	14.0a	102.347A	1164.52C
JN38-GmWRI14-4	43.52±1.5	12.71±1.1	12.01±1.5	41.56±1.3	32.88±3.1	13.40±1.7	30A	115A	19.29C	18.2a	105.429A	1142.20C
JN38-GmWRI14-5	45.85±1.6	13.27±1.6	12.06±1.7	42.84±1.3	31.30±1.8	12.06±0.9	31A	115A	19.459C	12.5a	101.280A	1014.4D
JN38-GmWRI14-6	44.33±1.5	13.36±1.2	10.96±0.7	40.18±4.1	30.61±2.2	12.29±1.4	29A	110A	29.79C	15.5a	104.220A	1022.24D
Range	146%-175%	146%-157%	273%-321%	23.2%-41.2%	11.4%-52.1%	198%-263%	-	-	176%-271%	-	-	241%-311%

Note: The different uppercase letters indicate significant differences at $P < 0.01$, the different lower letters indicate significant differences at $P < 0.05$, as determined by Duncan's multiple-range test
