# Supplementary figures and images for: Genome-Wide Association Study Identifies Candidate Genes Related to the Linoleic Acid Content in Soybean Seeds
Source: Int J Mol Sci. 2021 Dec 31;23(1):454. doi: 10.3390/ijms23010454 (PMC8745128; doi:10.3390/ijms23010454)

A

## Population Structure

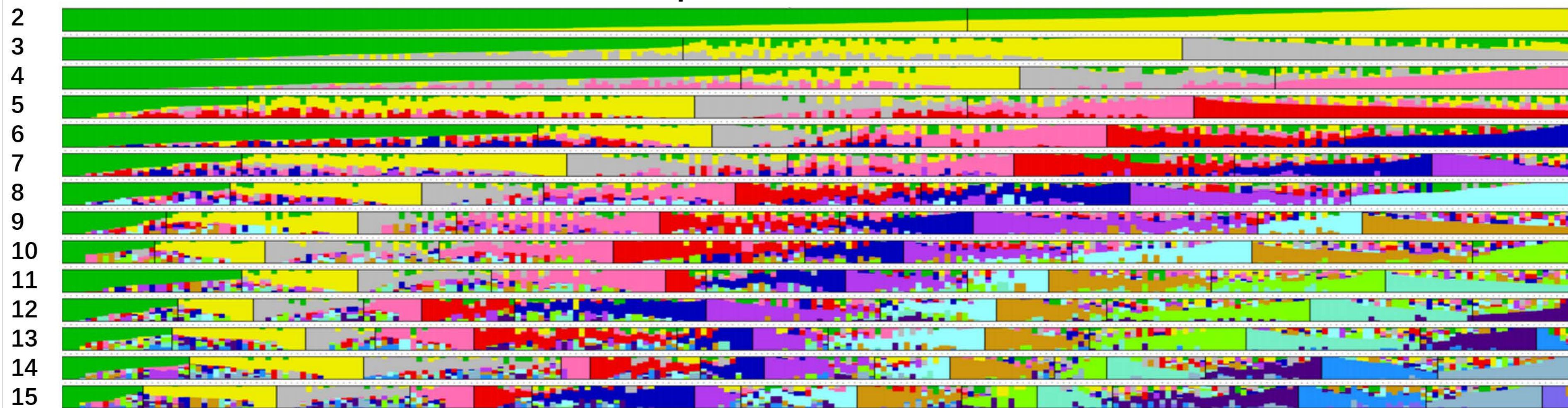

B

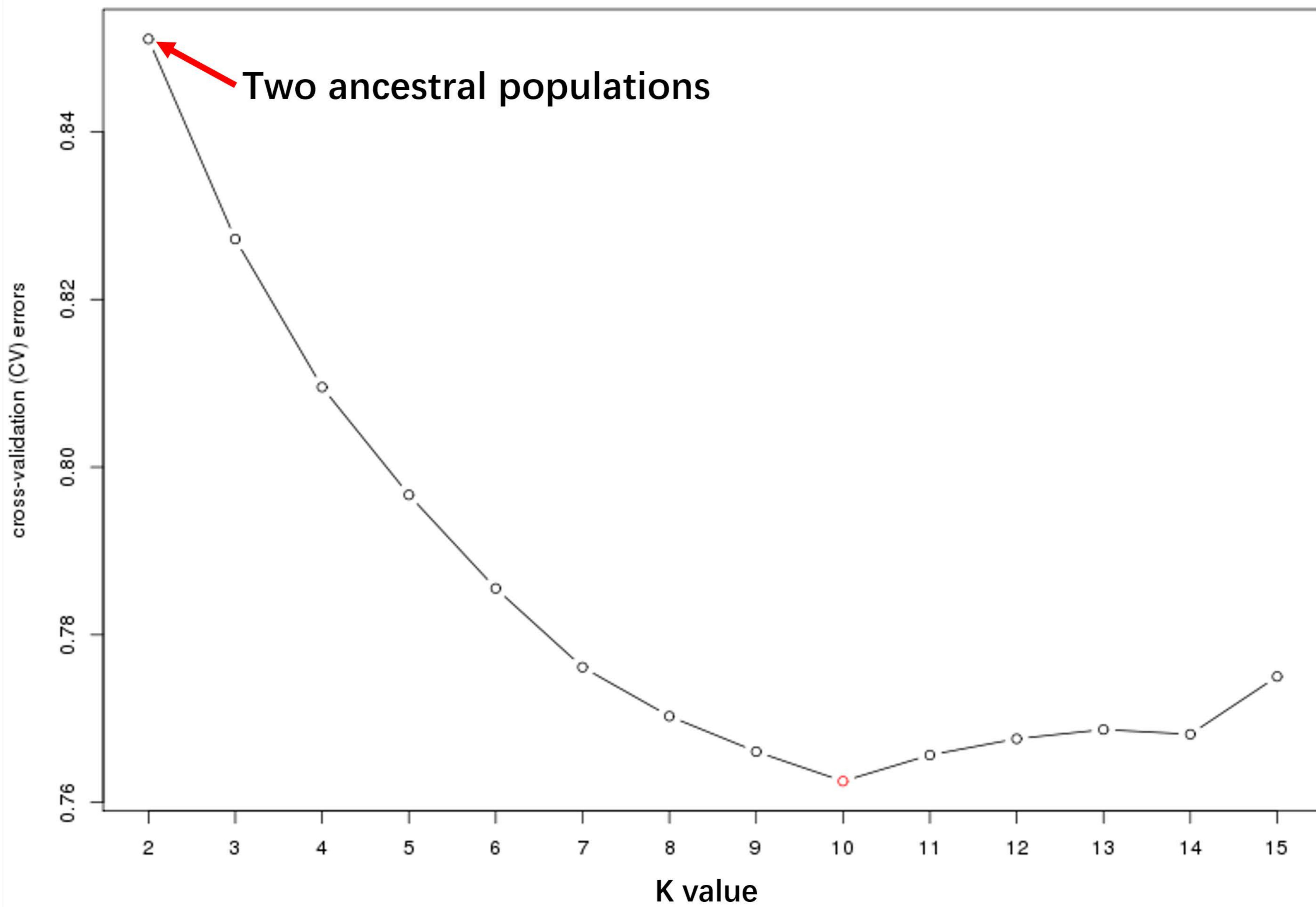

Supplement: Supplementary file 1 [file ijms-23-00454-s001.zip › Figure S1.pdf]

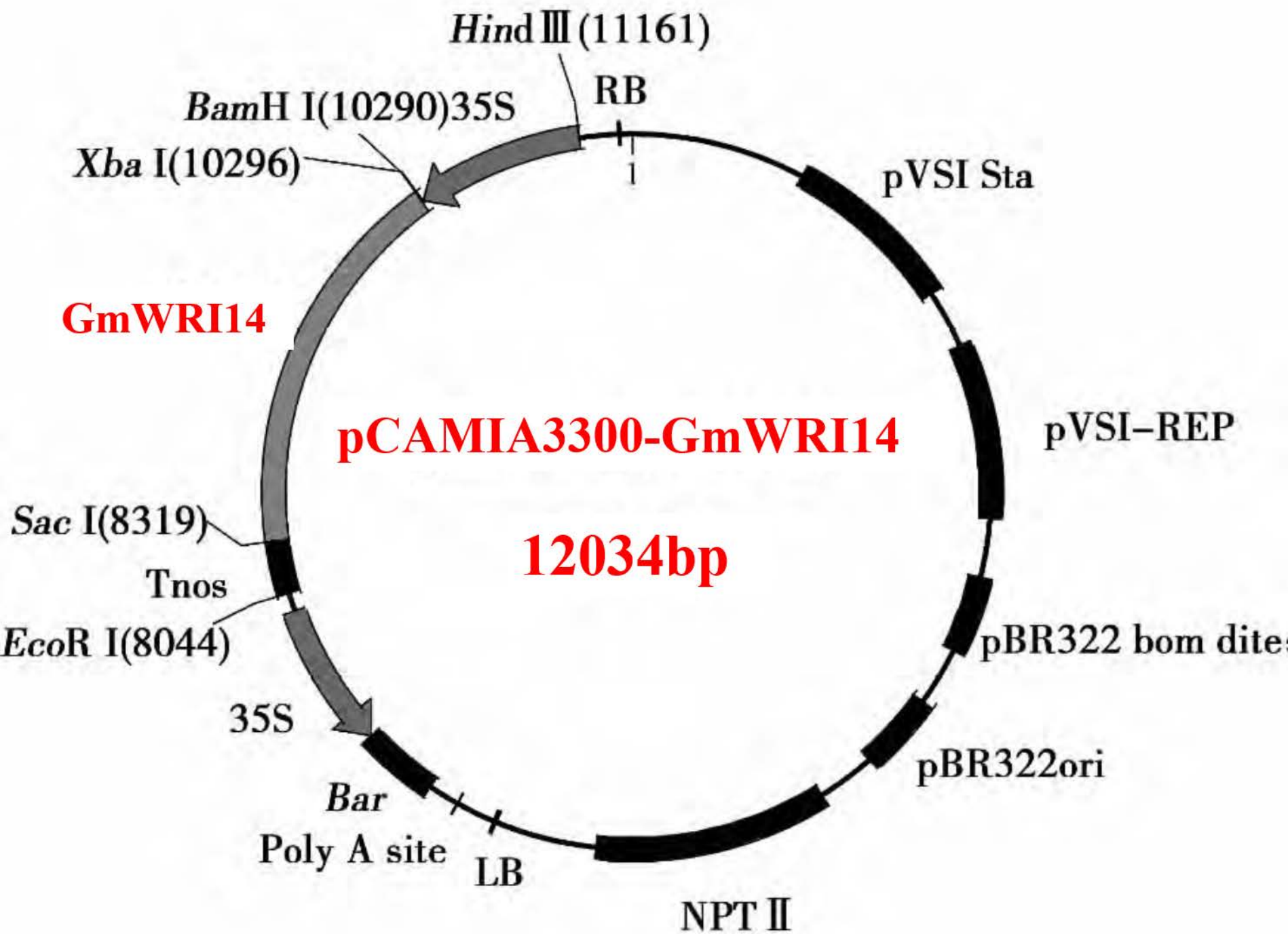

Supplement: Supplementary file 1 [file ijms-23-00454-s001.zip › Figure S3.pdf]
